# A New Cardiovascular Mock Loop Driven by Novel Active Capacitance in Normal and Abnormal Conditions

**DOI:** 10.1155/2023/2866637

**Published:** 2023-10-27

**Authors:** Mehmet Iscan, Aydin Yesildirek

**Affiliations:** Yildiz Technical University, Istanbul, Türkiye

## Abstract

The hybrid mock circulatory loop (hMCL) serves as a crucial hemodynamic simulation tool, offering exceptional flexibility, controllability, and reproducibility for investigating the mechanisms underlying cardiovascular diseases (CVD) in a controlled environment, circumventing the limitations of live organism studies. This paper introduces a novel design and control strategy for hMCL, introducing a novel left ventricle volume–elastance (LVVE) equation that unifies the autoregulation of the Frank–Starling mechanism (FSM) with left ventricle contractility (LVC). LVVE establishes a dynamic link between left ventricular volume (LVV) and LVC, inherently satisfying the regulatory relationship between left ventricular pressure (LVP) and LVV through a mathematical equation. For the first time, LVVE integration significantly enhances the physiological relevance of hMCL by faithfully replicating FSM responses across diverse conditions, including aortic stenosis (AS), variations in systemic vascular resistance (SVR), and heart rate (HR) variations. Furthermore, this study introduces the stability proofs for the discrete closed-loop hMCL, enabling real-time proportional valve control through discrete feedback linearization—an innovative departure from conventional methods. Notably, FSM emulation is achieved by tracking reference maximum and minimum LVV values, eliminating the reliance on predefined functions or existing data, such as the maximum LV elastance value. Rigorous experimental validation, encompassing numerical simulations and comparative analyses with prior research, attests to the precision and efficacy of the proposed hMCL in faithfully replicating both normal and abnormal CV conditions. Significantly, the hMCL demonstrates that increasing HR enhances LVC while maintaining physiological pressures; however, this increase in LVC corresponds with a decrease in LVV, in alignment with human data and FSM principles. Crucially, the coupling mechanism between the FSM and LVC yields results of enhanced physiological fidelity, significantly advancing the hMCL's utility in physiological research. Moreover, the hMCL's capacity to simulate critical cardiovascular scenarios, including AS, SVR fluctuations, and HR variations, underscores its versatility and substantial potential for investigating complex CV dynamics.

## 1. Introduction

The cardiovascular system (CVS) is the whole circulatory system for pumping blood across the body to sustain the gas and nutrients exchange through the tissues and cells [1]. Any dysfunction during the entire process is named cardiovascular disease (CVD). According to the World Health Organization (WHO), CVDs are the leading cause of death globally. In 2019, approximately 17.9 million people died due to CVDs [2]. In 2015, in Europe alone, 85 million people were affected by cardiovascular diseases. That same year, 3.9 million people lost their lives due to cardiovascular diseases. This emphasizes the ongoing need for research, prevention, and treatment efforts to address the global burden of CVDs [3]. Therefore, modeling, diagnosis, and curing of the CVDs have been one of the primary important research fields for medical and engineering applications. The CVS tests can almost be conducted in vivo or in vitro in which in vivo tests are performed within a living organism while in vitro tests are performed outside of a living organism. Therefore, in vitro tests and equipment have become increasingly important in cardiovascular (CV) research. While in vivo examinations offer high precision and real-time physiological data from living organisms, they are often subject to significant regulatory restrictions imposed by organizations like the FDA and NCBI, limiting their practicality [4, 5]. Besides, the repetition of the in vivo tests is challenging; testing different conditions is almost impossible or become high time consumed processes. However, when conducting experiments, in vitro testing offers a superior level of control over the working environment than animal testing. Specifically for the CVS, hydraulic components are affected by spurious effects, making it impossible for hydraulic components to imitate mathematical models of the system. So, for the sake of the reproducibility and the studying different conditions, in vitro tests and equipment become prominent.

To assist the CVS in cases of partial or complete dysfunction, especially pertaining to the heart, various types of devices are available. These include FDA-approved equipment such as artificial heart valves, stents, and ventricular assisted devices [6]. These systems range incomplexity, from simple pulse duplicators to feedback-controlled systems that integrate the contractile activity of the heart [7–9]. These systems are highly complicated to be tested in only in vitro case because the complicated dynamics cannot be involved into the in vitro setup due to the requirement of complex design. In order to test these equipments, the hybrid mock circulatory loop (hMCL), which combines both in vitro and in silico features, is commonly utilized for in vitro testing circulation systems and validation processes for these devices [10]. The hMCL enhances system flexibility and accuracy with the aid of the features of both mathematical model and physical device. Especially for the FrankStarling mechanism (FSM) which is the regulation between preload and cardiac output (CO), it is important to replicate its CV responses affecting the interaction between CV assist devices and the CVS. However, the hMCL lacking preload and afterload response fail to yield physiological results. Early researchers utilized the time-varying elastance model developed by Suga et al. [11] to incorporate the FSM, which remains frequently employed in the design of such hMCLs [12]. This model remains widely used for driving the heart contraction mechanism in CV mock loops. Therefore, it continues to serve as a common reference for constructing these kinds of hMCLs [11].

Various approaches have been proposed in the literature for the hMCL replicating the FSM [13–15]. It is indeed possible to assess ventricular systole using these approaches [16]. The elastance-based controller responds to changes in preload, afterload, and left ventricular contractility (LVC) in a manner similar to the normal heart, as demonstrated through simulations and tests conducted using MCL components. However, the maximum elastance value, that represents the LVC capability, remains constant. The utilization of elastance-based control in such loops has emerged as one of the most optimal approaches, as the elastance function is a time-dependent arbitrary function that allows for modifications in the LVC [17]. Furthermore, several studies have been reported in the literature on controlling elastance-based mock loops [17–22]. These studies employed the elastance function to derive pressure–volume diagrams for cardiac energetics. However, the elastance function solely generates the total left ventricle pressure (LVP), without distinguishing between the passive and active pressures of the heart muscle. This differentiation is crucial for capturing the realistic physiological features of the CVS. The contraction process of the left ventricle (LV) involves the excitation of muscles in the active part only, and this aspect should be considered significant.

In order to evaluate the performance of elastance-based controllers under different normal and abnormal CV conditions, there have been numerous studies reported in the literature on the hMCL. Ferrari et al. [7] investigated pathophysiological conditions by modifying Emax and Emin. They generated reference left ventricle flow (LVQ) and LVP in software to drive the hMCL [19]. However, there was inconsistency with physiological data in terms of arterial pressure drop. In their subsequent improvement, they incorporated a compliance formulation based on Young's modulus [23]. Although they achieved good results that matched physiological data, they did not assess the wave propagation of LVQ using this model. Colacino et al. [18] enhanced the elastance model by incorporating the viscoelastic properties of ventricular tissue, as well as the inertial effects of walls and intraventricular blood. They used left ventricular volume (LVV) as a reference signal to implement the FSM with their controller technique. They extensively tested and validated preload, afterload, and the FSM using human data from Guyton's study. However, their controller design did not include LVC to fully satisfy the autoregulation of the FSM. Additionally, they used a gear pump to supply appropriate LVV, but the mathematical stability of the controller structure was not proven. Gregory et al. [24] designed MCL that replicated the left and right FSM responses and tested changes in preload using a controller that applied pressure references to a pneumatic actuator. However, they did not demonstrate the stability of driving pneumatic actuators, and the utilization of the LVP reference did not entirely reflect the time response of the autoregulation of the FSM. Janse-Park et al. [25] achieved preload sensitivity, regulation of stroke volume with mean arterial pressure, and baroreflex mechanisms by varying LVC using Emax. They did not define the FSM based on time-varying elastance but instead utilized an adaptive control approach without guaranteeing stability. Furthermore, the direct supply of LVQ using a gear pump was inconsistent with the physiological properties of the CVS. Mirzaei et al. [26] developed a convergence technique to simulate stenosis in the CVS. Although their results were promising, the convergence process did not allow for real-time control and application. Gregory et al. [27] constructed systemic, pulmonary, cerebral, and coronary circulation with autoregulation responses by validating human data. They introduced pneumatic control with the FSM responses of the ventricles, but stability was not guaranteed. However, repeatability was problematic for validating their results due to the absence of controlled dynamics. Salesch et al. [28] developed a closed-circuit hMCL to replicate the physiological behavior of the CVS. They controlled both aortic and left ventricular pressures but did not guarantee stability. They were the first to define the ESPVR and EDPVR of the LV to drive the system, especially for parameter estimation of the CVS. Vignali et al. [29] included the aortic complex using a 3D printed hMCL that simulated both aortic and mitral flows. The aortic flow waveform was used as an input, and supra-aortic vessel flow was accurately represented based on the literature. However, compliance control was lacking in the chambers, and the LVP profile was used to drive the hMCL. The entire system was controlled, and there were no free parameters to describe the passivity of the CVS. Linear approximation of LVC was used to emulate the time responses of the CVS, and nonlinear approximation was performed to improve this feature [12]. However, the relationship between elasticity and volume had not been studied to satisfy both the autoregulation of the FSM and the physiological pressure range in the LV section. Rocchi et al. [30] reviewed personalized MCL designs to provide physiological properties of the heart, especially in 3D models. They designed heart valves to represent aortic flow and pressure characteristics, but they did not incorporate the coupling of the LVC to provide the FSM. Furthermore, the stiffness of the LV chamber was a vital key parameter for replicating more consistent results that matched physiological human data. Rapp et al. [31] constructed an hMCL to simulate valvular stenosis and cardiac arrhythmia using a ventricular assisted device. However, the inputs of the system, such as LVP, VAD flow rate, and right atrium pressure, were not physiologically correct for the passive parameters. Packy et al. [32] developed MCL to characterize normovolemia, cardiogenic shock, and hyperdynamic circulation. Although the results were consistent with human data, the LV viscoelastic behavior and other tanks were manually controlled by using a syringeto set the proper compliance value, which posed difficulties in real-time application. Bardi et al. [33] proposed an hMCL to simulate hemodynamics in terms of 3D geometry and inlet/outlet conditions. However, the tank pressures were controlled, and compliance and resistance values needed to be set when the system was closed rather than during operation. Additionally, a stability rule was not provided for the gas valve to control the pressure values of the chambers.

Furthermore, it is crucial to incorporate gas modeling in the hMCL to capture the electrical and mechanical feature of the LVC resulting from the electrical activation of the heart and the filling of blood into the LV. Vandenberghe et al. [34] conducted a study highlighting that the intricate hemodynamic behavior of a mechanically assisted LV cannot be adequately captured by the time-varying elastance theory alone. Therefore, the development of a new LV model is necessary to accurately represent the interaction between the LVC and FSM. [34]. The novelty of this proposed method lies in the incorporation of capacitance and volume control equations to simulate the gas tank dynamics, forming the foundation of the proposed system. In this study, a new design and control strategy for a hMCL are proposed utilizing a novel LVV volume–elastance (LVV–E) equation which couples the properties of the LVC and FSM, aligning with physiological human data. Importantly, for the first time, LVC is derived from the physical gas chamber, representing the active component of the LV. Additionally, a discrete feedback linearization controller is designed to ensure stability in the gas valves, which serve as the sole inputs to our proposed system. Furthermore, the pathological conditions are simulated by manipulating aortic and systemic resistance values and validated using human data and reference models. Various heart rates and their associated cardiac outputs are demonstrated to verify the functionality of the FSM. The main contributions of the proposed study are: (1) replication of the FSM satisfying LVC; (2) discrete formulation of nonlinear dynamics of the CVS matching the proposed in vitro setup; (3) guaranteeing the stability of the closed-loop hMCL in discrete domain for real-time application; (4) eliminating the requirements for predefined functions or physiological data to emulate CVS; (5) utilizing one controlled parameter while the rest of the mare free to get more realistic results in CVS.

## 2. Materials and Methods

In this section, we provide a comprehensive overview of the proposed hybrid mock loop. First, we will present a general description of the system. Next, we will develop the state–space representation of the entire dynamics and provide a detailed derivation of the volume–elastance relationship. To ensure stability, we will established a discrete feedback linearization control approach for the gas valve.

### 2.1. The Proposed Hybrid Mock Circulatory Loop

**Figure 1:**
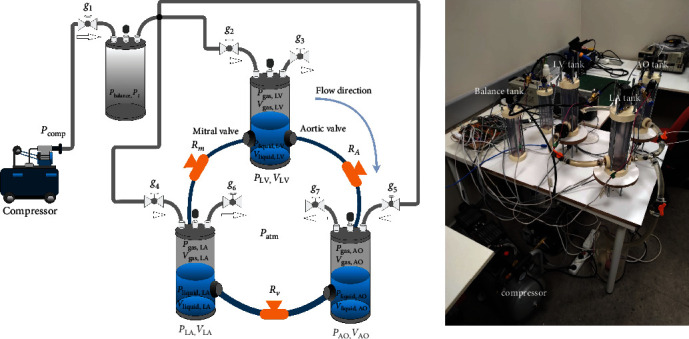
Mechanical structure of mock loop design.

The hMCL serves as a hydraulic test bench that facilitates the analysis of prosthetic functionality, design, and their interactions with the simulated CVS [11]. The key components of the CVS, including the left ventricle (LV), left atrium (LA), and aorta, are represented by physical tanks comprising both gas and liquid components. While the complete CVS consists of various compartments such as arteries, right ventricle, atrium, and capillaries, for specific CVS tests, a simplified geometry is employed, focusing on the essential compartments. In the proposed design of the hMCL, the LA, LV, and aorta compartments are modeled using physical tanks, as illustrated in Figure 1.

Figure 1 illustrates the complete configuration of the hMCL, comprising the LV, aorta, and LA tanks, with the liquid components depicted in blue. The LV tank is equipped with two distinct liquid valves: the mitral valve, responsible for transferring the liquid from the LA to the LV during the diastole phase, and the aortic valve, facilitating flow from the LV to the aorta tank during the systole phase. The construction of the entire platform involves three pipelines that connect each tank to specific adjustable resistances referred to as RM, RA, and RV. These resistances can be modified by increasing or decreasing their values to replicate pathophysiological scenarios in the hMCL.

To account for the naturally low-pressure characteristics of the LV chamber, a balance tank (Tank1) is employed to reduce the supplied gas pressure. Within the pipeline, a valve is included to decrease the pressure, as it is more convenient to apply control mechanisms at lower pressure levels. The hMCL incorporates proportional gas valves to regulate gas inlet and outlet, and these valves are controlled by adjusting the electrical current based on the pressure difference balance between interconnected tanks.

To regulate the gas inlet and outlet, proportional gas valves have been incorporated into the hMCL system. These valves can be controlled by adjusting the electrical current in response to the pressure difference balance between interconnected tanks. The system consists of three valves: g1, which is situated between the compressor and Tank 1; g2, located between Tank 1 and the LV gas section; and g3, which connects the left ventricle gas section to the atmosphere.

**Figure 2:**
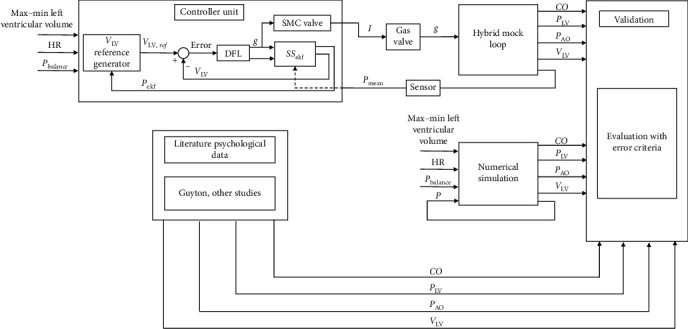
Functional block diagram of the proposed system.

In order to explain the whole processes, the functional block diagram is illustrated in Figure 2.

Figure 2 describes both the numerical and hMCL functional diagrams, illustrating the implementation of physical and numerical processes. The reference signals chosen for the system are heart rate and the maximum and minimum values of LVV. The hMCL and numerical simulations generate outputs such as cardiac output (CO), LVV, LVP (Plv), and aortic pressure (Pao). Furthermore, physiological human data from the literature are used to validate the performance of the proposed hMCL. In the hMCL, the control algorithm is implemented on anSTM32F746 microprocessor, incorporating both the system's dynamic representation of the numerical model and the discrete feedback linearization algorithm. To suppress the measurement noise, an extended Kalman filter (EKF) is employed in the controller unit, as described in the subsection of Figure 2. The driving current is calculated using the SMC valve flow-current–pressure diagram, which converts the controller input (opening rate, g) into the appropriate current value. The principle of operation of the hMCL described above and how it is carried out can be found in the video link [35].

In the controller design, the error is computed by subtracting the generated LVV reference from the measured one. This error value is then fed into the discrete feedback linearization equation to obtain the controller input. Additionally, an EKF is established by linearizing the total state–space representation. Noisy pressure measurements are inputted into this filter, and the filtered pressure values are used to calculate the appropriate controller input.

### 2.2. System Dynamic Representation

In this section, we established the dynamic representation of the current hMCL system by deriving the equations of the motion. These equations include the conservation of mass rule and the capacitance equation. Furthermore, we provide a comprehensive list of symbols related to the equations in Table 1.

### 2.3. System Dynamic Representation

The mock loop representing the dynamics of the left ventricular of the cardiovascular loop consists of three tanks, LV, LA, and AO for the fluid motion and the balance tank to produce the required pressures as depicted in Figure 1. In order to account for variable capacitance, the dynamics of each tank need to be derived based on the inlet and outlet flows. The dynamics are expressed in compliance form, incorporating the mass rule for both gas and liquid flows. Additionally, the conversion of gas density into pressure is achieved by applying the ideal gas law to each tank. The LV, aorta, and LA tanks are modeled considering the gas–liquid interaction and conservation of mass law, whereas the Tank 1 represents only the gas component of this dynamic system. The general conservation of the total mass in a tank is given as follows:

(1) $$\begin{array}{c} {\dot{m}}_{st,i}={\dot{m}}_{i_{\text{gas in }}}-{\dot{m}}_{i_{\text{gas out }}}+{\dot{m}}_{i_{\text{liquid in }}}-{\dot{m}}_{i_{\text{liquid out }}}, \end{array}$$

where *i* = (LV, AO, LA) the tank index and the fluid resistances are modeled as follows:

(2) $$\begin{array}{c} \begin{array}{c} {\dot{m}}_{i_{\text{in }}}=g_{i_{in}}\Delta P_{i_{\text{in }}}\\ {\dot{m}}_{i_{\text{out }}}=g_{i_{\text{out }}}\Delta P_{i_{\text{out }}}, \end{array} \end{array}$$

with *m*_*i*_, Δ*P*_*i*_, and *g*_*i*_=1/*R*_*i*_ the mass, the pressure difference, and the conductance rates of the component i as shown in Table 1, respectively. On the other hand, the rate of change in the stored fluid mass is written in terms of its volume and density as follows:

(3) $$\begin{array}{c} {\dot{m}}_{st,i}={\dot{\rho }}_{i}V_{i}+\rho_{i}{\dot{V}}_{i}, \end{array}$$

with *ρ*_*i*_ fluid density for *i* as given in Table 1. Therefore, the rate of change in density, when exists, is driven by substituting Equation (3) into Equation (1) as follows:

(4) $$\begin{array}{c} {\dot{\rho }}_{i}=\frac{1}{V_{i}}\left[\left({\dot{m}}_{i_{\text{gas in }}}-{\dot{m}}_{i_{\text{gas out }}}+{\dot{m}}_{i_{\text{liquid in }}}-{\dot{m}}_{i_{\text{liquid out }}}\right)-\rho_{i}{\dot{V}}_{i}\right]. \end{array}$$

This enables us to express the variable capacitance phenomenon. For our system the following assumptions are made

- *V*_*T*_ is constant,
- The temperature is constant,
- The shape of tank is rigid.

the time derivative of the pressure, *P*_*i*_=*ρ*_*i*_*RT*, becomes

(5) $$\begin{array}{c} {\dot{P}}_{i}={\dot{\rho }}_{i}RT, \end{array}$$

with *R* the specific gas constant, *T* temperature. Combining Equations (4) and (5) represents the equation of motion for the tank “*i*”.

Using Equations (4) and (5), the general pressure law for each tank can be written as follows:

(6) $$\begin{array}{c} {\dot{P}}_{i}=\frac{RT}{V_{i}}\left[\left({\dot{m}}_{i_{\text{gas in }}}-{\dot{m}}_{i_{\text{gas out }}}+{\dot{m}}_{i_{\text{liquid in }}}-{\dot{m}}_{i_{\text{liquid out }}}\right)-\frac{P_{i}}{RT}{\dot{V}}_{i}\right]. \end{array}$$

In the next sections, we will develop equations of motion for each tank, individually.

### 2.4. Modeling the Balance Tank

The mass flow rate of the balance tank defined by the difference between in/out gas flow rates is given in

(7) $$\begin{array}{c} {\dot{m}}_{st,1}={\dot{m}}_{1}-{\dot{m}}_{2}, \end{array}$$

with *m*_*i*_ the rate of changes in in/out masses that may, further, be expressed as follows:

(8) $$\begin{array}{c} \begin{array}{c} {\dot{m}}_{1}=g_{1}\left(P_{c}-P_{1}\right)\\ {\dot{m}}_{2}=g_{2}\left(P_{1}-P_{\text{LV},g}\right), \end{array} \end{array}$$

where the conductance rates, *g*_*i*_, and the pressure differences, Δ*P*_*i*_, for *i* = 1, 2. Since the stored gas mass is

(9) $$\begin{array}{c} m_{st,1}=\rho_{1}V_{1}, \end{array}$$

and taking its derivative yields

(10) $$\begin{array}{c} {\dot{m}}_{st,1=}{\dot{\rho }}_{1}V_{1}. \end{array}$$

Notice that using Equation (5), the mass flow rate can also be written as follows:

(11) $$\begin{array}{c} {\dot{m}}_{st,1}=\frac{V_{1}}{RT}{\dot{P}}_{1}, \end{array}$$

where *V*_1_/*RT* is the capacitance *C*_1_=1/*α*_1_. Thus, the pressure dynamics becomes

(12) $$\begin{array}{c} {\dot{P}}_{1}=\alpha_{1}\left\{g_{1}\left(P_{c}-P_{1}\right)-g_{2}\left(P_{1}-P_{\text{LV},g}\right)\right\}, \end{array}$$

with the elastance *α*_1_ as given in Table 1.

### 2.5. Modeling the Balance Tank

LV tank is composed of two parts: the gas and liquid sections in which the former pressurizes the LV tank and the latter is used to transfer liquid according to the CVS. The conservation of mass for the tank for gas section is used to define the equations of motion as follows:

(13) $$\begin{array}{c} {\dot{m}}_{\text{LV},g}={\dot{m}}_{2}-{\dot{m}}_{3}, \end{array}$$

where the intake and outtake masses flow rates

(14) $$\begin{array}{c} \begin{array}{c} {\dot{m}}_{2}=g_{2}\left(P_{1}-P_{\text{LV},g}\right)\\ {\dot{m}}_{3}=g_{3}\left(P_{\text{LV},g}-P_{\text{out }}\right), \end{array} \end{array}$$

with *g*_2_ and *g*_3_ are the inlet and outlet conductances of LV chamber given in Table 1. *P*_out_ is the atmosphere pressure. Similarly, the storage mass can be described using Equation (3) yielding

(15) $$\begin{array}{c} {\dot{m}}_{\text{LV},g}={\dot{C}}_{\text{LV},g}P_{\text{LV},g}+C_{\text{LV},g}{\dot{P}}_{\text{LV},g}, \end{array}$$

or

(16) $$\begin{array}{c} {\dot{m}}_{\text{LV},g}={\dot{\rho }}_{\text{LV},g}V_{\text{LV},g}+\rho_{\text{LV},g}{\dot{V}}_{\text{LV},g}, \end{array}$$

with *C*_LV,*g*_ and *ρ*_LV,*g*_ are the gas capacitance and the gas density in the LV tank, respectively. In Equations (15) and (16), we can write the storage of mass in terms of either capacitance or density forms. Using Equations (5) and (16), we get Equation (17).

(17) $$\begin{array}{c} {\dot{m}}_{\text{LV},g}={\dot{P}}_{\text{LV},g}\frac{V_{\text{LV},g}}{RT}+P_{\text{LV},g}\frac{{\dot{V}}_{\text{LV},g}}{RT}. \end{array}$$

Equation (17) is utilized to obtain specific compliance value on gas section since physiological LV model changes its capacitance value when contraction/relaxation happens. The gas capacitance of LV chamber is *C*_LV,*g*_=*V*_LV,*g*_/*RT*, then

(18) $$\begin{array}{c} {\dot{V}}_{\text{LV},g}/RT={\dot{C}}_{\text{LV},g}. \end{array}$$

Using the proposed hMCL assumptions given above, the total tank volume (*V*_*T*_) is expressed by Equation (19) as follows:

(19) $$\begin{array}{c} V_{T}=V_{\text{LV},g}+V_{\text{LV},l}\to V_{\text{LV},g}=V_{T}-V_{\text{LV},l}{\dot{V}}_{\text{LV},g}/RT={\dot{C}}_{\text{LV},g}, \end{array}$$

with *V*_LV,*g*_ and *V*_LV,*l*_ are gas and liquid volume of LV chamber. The liquid volume of LV iswritten using Equation (19) like

(20) $$\begin{array}{c} V_{\text{LV},l}=Ay\to V_{\text{LV},l}=A\left(\frac{P_{\text{LV}}-P_{\text{LV},g}}{\rho_{l}g}\right), \end{array}$$

where *y*, *A*, *P*_LV_, and *P*_LV,*g*_ are liquid head, area, the total and gas pressure of LV chamber, respectively.

The *C*_LV,*g*_ is written using Equation (18) in terms of the pressures of LV chamber

(21) $$\begin{array}{c} C_{\text{LV},g}=\frac{1}{RT}\left[V_{T}-\frac{A}{\rho_{l}g}\left(P_{\text{LV}}-P_{\text{LV},g}\right)\right]. \end{array}$$

Taking the derivative of Equation (19) yields

(22) $$\begin{array}{c} {\dot{V}}_{T}={\dot{V}}_{\text{LV},l}+{\dot{V}}_{\text{LV},g}\to {\dot{V}}_{\text{LV},g}=-{\dot{V}}_{\text{LV},l}, \end{array}$$

where *V*_LV,*g*_ and *V*_LV,*l*_ are the gas and liquid volume of LV chamber. From the conversation of the mass the volume dynamics is derived using Equation (4) as

(23) $$\begin{array}{c} {\dot{V}}_{\text{LV},l}=\frac{1}{\rho_{l}}\left[g_{TLI}\left(t\right)\left(P_{\text{LA}}-P_{\text{LV}}\right)-g_{TLO}\left(t\right)\left(P_{\text{LV}}-P_{AO}\right)\right], \end{array}$$

where *ρ*_*l*_ are the density of liquid, the time-varying mitral and aortic conductances of *g*_*TLI*_(*t*) and *g*_*TLO*_(*t*) as shown by Iscan and Yesildirek [36]. The liquid mass flow rates are now put into Equation (23). The definition of those conductances are

(24) $$\begin{array}{c} \begin{array}{c} g_{TLI}\left(t\right)=\theta_{m}\left(t\right)\left[g_{m}+g_{M}+g_{p}\right]\\ g_{TLO}\left(t\right)=\theta_{AO}\left(t\right)\left[g_{AO}+g_{AO}+g_{p}\right], \end{array} \end{array}$$

where *θ*_*m*_(*t*) and *θ*_*AO*_(*t*) are the opening rates of the mitral and aortic valves, respectively. *g*_*i*_ are the pipeline and valve conductance rates given in Table 1. Using Equations (18) and (23) gives us the time derivative of the LV gas capacitance formulation as follows:

(25) $$\begin{array}{c} {\dot{C}}_{\text{LV},g}=\frac{1}{\rho_{l}RT}\left[g_{TLO}\left(t\right)\left(P_{\text{LV}}-P_{AO}\right)-g_{TLI}\left(t\right)\left(P_{\text{LA}}-P_{\text{LV}}\right)\right], \end{array}$$

with *P*_LA_ and *P*_*AO*_ are total pressure of LA and aorta chamber. Using Equation (15), we obtain

(26) $$\begin{array}{c} {\dot{P}}_{\text{LV},g}=-\frac{{\dot{C}}_{\text{LV},g}}{C_{\text{LV},g}}P_{\text{LV},g}+\frac{1}{C_{\text{LV},g}}\left[g_{2}\left(P_{1}-P_{\text{LV},g}\right)-g_{3}\left(P_{\text{LV},g}-P_{\text{out }}\right)\right]. \end{array}$$

Now, the total pressure, LVP, is expressed by Equation (27) as follows:

(27) $$\begin{array}{c} {\dot{P}}_{\text{LV}}={\dot{P}}_{\text{LV},g}+\rho_{l}G\frac{{\dot{V}}_{\text{LV},l}}{A}, \end{array}$$

with *G* is the gravity of the earth. Putting both Equations (26) and (23) yields Equation (28) as follows:

(28) $$\begin{array}{c} \begin{array}{c} {\dot{P}}_{\text{LV}}=-\frac{{\dot{C}}_{\text{LV},g}}{C_{\text{LV},g}}P_{\text{LV},g}+\frac{1}{C_{\text{LV},g}}\left[g_{2}\left(P_{1}-P_{\text{LV},g}\right)-g_{3}\left(P_{\text{LV},g}-P_{\text{out }}\right)\right]\\ +\frac{g}{A}\left[g_{TLI}\left(t\right)\left(P_{\text{LA}}-P_{\text{LV}}\right)-g_{TLO}\left(t\right)\left(P_{\text{LV}}-P_{AO}\right)\right]. \end{array} \end{array}$$

It is important to note that *θ*_*m*_(*t*) and *θ*_*AO*_(*t*) are equal to “0” when the opening angle is lower than 45° as stated by Iscan and Yesildirek [36].

### 2.6. Modeling the Aorta Tank

In the aorta tank, we have total mass equation, *m*_*AO*_=*C*_*AO*,*g*_*P*_*AO*,*g*_ and taking derivative of it yielding

(29) $$\begin{array}{c} {\dot{m}}_{AO,g}={\dot{C}}_{AO,g}P_{AO,g}+C_{AO,g}{\dot{P}}_{AO,g}, \end{array}$$

where *C*_*AO*,*g*_ and *P*_*AO*,*g*_ are the gas capacitance and pressure of the aorta chamber. The inlet and outlet gas flow are defined by both Equations (30) and (31).

(30) $$\begin{array}{c} {\dot{m}}_{AO,g}={\dot{m}}_{5}-{\dot{m}}_{7}, \end{array}$$

where

(31) $$\begin{array}{c} \begin{array}{c} {\dot{m}}_{5}=g_{5}\left(P_{1}-P_{AO,g}\right)\\ {\dot{m}}_{7}=g_{7}\left(P_{AO,g}-P_{out}\right), \end{array} \end{array}$$

where *g*_5_ and *g*_7_ are the gas inlet and outlet conductance rate of aorta chamber. Using *C*_*AO*,*g*_=*V*_*AO*,*g*_/*RT* given in Equation (18), we take the derivative of gas capacitance of aorta tank like

(32) $$\begin{array}{c} {\dot{C}}_{AO,g}=\frac{{\dot{V}}_{AO,g}}{RT}. \end{array}$$

Then both aortic gas capacitance and derivative of it are given in Equations (33) and (34) as follows:

(33) $$\begin{array}{c} C_{AO,g}=\frac{1}{RT}\left[V_{T}-\frac{A}{\rho_{l}G}\left(P_{AO}-P_{AO,g}\right)\right], \end{array}$$

(34) $$\begin{array}{c} {\dot{C}}_{AO,g}=\frac{1}{\rho_{l}RT}\left[g_{SYS}\left(P_{AO}-P_{LA}\right)-g_{TLO}\left(t\right)\left(P_{LV}-P_{AO}\right)\right], \end{array}$$

with *g*_*SYS*_ is total systemic conductance rate and equals to *g*_*SYS*_=*g*_*sys*_+*g*_*P*_ from Table 1.

The aortic gas dynamic is now written like

(35) $$\begin{array}{c} {\dot{P}}_{AO,g}=-\frac{{\dot{C}}_{AO,g}}{C_{AO,g}}P_{AO,g}+\frac{1}{C_{AO,g}}\left[g_{5}\left(P_{1}-P_{AO,g}\right)g_{7}\left(P_{AO,g}-P_{\text{out }}\right)\right]. \end{array}$$

Now, ${\dot{P}}_{AO}=\rho_{l}G{\dot{V}}_{AO,l}/A$ then using Equation (35) with ${\dot{P}}_{AO}={\dot{P}}_{AO,l}+{\dot{P}}_{AO,g}$ we get

(36) $$\begin{array}{c} \begin{array}{c} {\dot{P}}_{AO}=-\frac{{\dot{C}}_{AO,g}}{C_{AO,g}}P_{AO,g}+\frac{1}{C_{AO,g}}\left[g_{5}\left(P_{1}-P_{AO,g}\right)-g_{7}\left(P_{AO,g}-P_{\text{out }}\right)\right]\\ +\frac{G}{A}\left[g_{TLO}\left(t\right)\left(P_{LV}-P_{AO}\right)-g_{SYS}\left(P_{AO}-P_{LA}\right)\right]. \end{array} \end{array}$$

### 2.7. Modeling the Left Atrium Tank

In the LA tank, the conversation of mass is written by *m*_LA,*g*_=*C*_LA,*g*_*P*_LA,*g*_ and taking derivative gives us

(37) $$\begin{array}{c} {\dot{m}}_{\text{LA},g}={\dot{C}}_{\text{LA},g}P_{\text{LA},g}+C_{\text{LA},g}{\dot{P}}_{\text{LA},g}, \end{array}$$

where *C*_LA,*g*_ and *P*_LA,*g*_ are the gas capacitance and pressure of LA chamber. The inlet and outlet gas flow are defined by both Equations (38) and (39) as follows:

(38) $$\begin{array}{c} {\dot{m}}_{\text{LA},g}={\dot{m}}_{4}-{\dot{m}}_{6}, \end{array}$$

where

(39) $$\begin{array}{c} \begin{array}{c} {\dot{m}}_{4}=g_{4}\left(P_{1}-P_{\text{LA},g}\right)\\ {\dot{m}}_{6}=g_{6}\left(P_{\text{LA},g}-P_{\text{out}}\right), \end{array} \end{array}$$

with *g*_4_ and *g*_6_ are the gas inlet and outlet conductance rate given in Table 1. Using $C_{\text{LA},g}=\frac{V_{\text{LA},g}}{RT},$ we obtain

(40) $$\begin{array}{c} {\dot{C}}_{\text{LA},g}=\frac{{\dot{V}}_{\text{LA},g}}{RT}, \end{array}$$

where *V*_LA,*g*_ is the gas volume of LA chamber. Now both LA gas capacitance and derivative of it can be stated in Equations (41) and (42).

(41) $$\begin{array}{c} C_{\text{LA},g}=\frac{1}{RT}\left[V_{T}-\frac{A}{\rho_{l}g}\left(P_{\text{LA}}-P_{\text{LA},g}\right)\right], \end{array}$$

(42) $$\begin{array}{c} {\dot{C}}_{\text{LA},g}=\frac{1}{\rho_{l}RT}\left[g_{TLI}\left(t\right)\left(P_{\text{LA}}-P_{\text{LA}}\right)-g_{SYS}\left(P_{AO}-P_{\text{LA}}\right)\right]. \end{array}$$

The LA gas dynamic is now expressed with

(43) $$\begin{array}{c} {\dot{P}}_{\text{LA},g}=-\frac{{\dot{C}}_{\text{LA},,g}}{C_{\text{LA},g}}P_{\text{LA},g}+\frac{1}{C_{\text{LA},g}}\left[g_{4}\left(P_{1}-P_{\text{LA},g}\right)-g_{6}\left(P_{\text{LA},g}-P_{\text{out }}\right)\right]. \end{array}$$

Now ${\dot{P}}_{\text{LA},l}=\rho_{l}G{\dot{V}}_{\text{LA},l}/A$ using Equation (43) with ${\dot{P}}_{\text{LA}}={\dot{P}}_{\text{LA},l}+{\dot{P}}_{\text{LA},g}$, we obtain

(44) $$\begin{array}{c} \begin{array}{c} {\dot{P}}_{LA}=-\frac{{\dot{C}}_{LA,g}}{C_{LA,g}}P_{LA,g}+\frac{1}{C_{LA,g}}\left[g_{4}\left(P_{1}-P_{LA,g}\right)-g_{6}\left(P_{LA,g}-P_{\text{out }}\right)\right]\\ +\frac{g}{A}\left[g_{SYS}\left(P_{AO}-P_{LA}\right)-g_{LTI}\left(t\right)\left(P_{LA}-P_{LV}\right)\right]. \end{array} \end{array}$$

After that, the equations of motion can be rewritten as a state–space representation form which is illustrated in Equation (45).

(45) $$\begin{array}{c} \dot{x}=A\left(x,t\right)x+B\left(x\right)u, \end{array}$$

(46) $$\begin{array}{c} \begin{array}{c} x=\left[\begin{array}{c} P_{1}\\ P_{\text{LA},g}\\ P_{\text{LA}}\\ P_{AO,g}\\ P_{AO}\\ P_{\text{LA},g}\\ P_{\text{LA}} \end{array}\right]u=\left[\begin{array}{c} g_{1}\\ g_{2}\\ g_{3}\\ g_{4}\\ g_{5}\\ g_{6}\\ g_{7} \end{array}\right]\\ A=\left[\begin{array}{cccc} 0 & 0 & 0 & 0\\ 0 & -\frac{{\dot{C}}_{\text{LV},g}\left(x\right)}{C_{\text{LV},g}\left(x\right)} & 0 & 0\\ 0 & -\frac{{\dot{C}}_{\text{LV},g}\left(x\right)}{C_{\text{LV},g}\left(x\right)} & \frac{g}{A}\left(-g_{TLI}\left(t\right)-g_{TL0}\left(t\right)\right) & 0\\ 0 & 0 & 0 & -\frac{{\dot{C}}_{AO,g}\left(x\right)}{C_{AO,g}\left(x\right)}\\ 0 & 0 & \frac{g}{A}g_{TL0}\left(t\right) & -\frac{{\dot{C}}_{AO,g}\left(x\right)}{C_{AO,g}\left(x\right)}\\ 0 & 0 & 0 & 0\\ 0 & 0 & \frac{g}{A}g_{TLI}\left(t\right) & 0 \end{array}\right.\ldots \\ \left.\begin{array}{ccc} 0 & 0 & 0\\ 0 & 0 & 0\\ \frac{g}{A}g_{TL0}\left(t\right) & 0 & \frac{g}{A}g_{TLI}\left(t\right)\\ 0 & 0 & 0\\ \frac{g}{A}\left(-g_{TL0}\left(t\right)-g_{SYS}\right) & 0 & \frac{g}{A}g_{SYS}\\ 0 & -\frac{{\dot{C}}_{\text{LA},g}\left(x\right)}{C_{\text{LA},g}\left(x\right)} & 0\\ \frac{g}{A}g_{SYS} & -\frac{{\dot{C}}_{\text{LA},g}\left(x\right)}{C_{\text{LA},g}\left(x\right)} & \frac{g}{A}\left(-g_{SYS}-g_{TLI}\left(t\right)\right) \end{array}\right]\\ B=\left[\begin{array}{ccc} \left(P_{c}-P_{1}\right)\alpha_{1} & -\left(P_{1}-P_{\text{LV},g}\right)\alpha_{1} & 0\\ 0 & \frac{1}{C_{\text{LV}}g\left(x\right)}\left(P_{1}-P_{\text{LV},g}\right) & -\frac{1}{C_{\text{LV}}g\left(x\right)}\left(P_{\text{LV},g}-P_{\text{out }}\right)\\ 0 & \frac{C_{\text{LV},g}\left(x\right)}{C_{L}}\left(P_{1}-P_{\text{LV},g}\right) & -\frac{C_{\text{LV},g}\left(x\right)}{C_{\text{LV},g}}-\left.P_{\text{out }}\right)\;\ldots \\ 0 & 0 & 0\\ 0 & 0 & 0\\ 0 & 0 & 0\\ 0 & 0 & 0 \end{array}\right.\\ \left.\begin{array}{cccc} 0 & 0 & 0 & 0\\ 0 & 0 & 0 & 0\\ 0 & 0 & 0 & 0\\ \frac{1}{C_{AO_{1}g}\left(x\right)}\left(P_{1}-P_{AO,g}\right) & 0 & -\frac{1}{C_{AO_{1}g}\left(x\right)}\left(P_{AO,g}-P_{\text{out }}\right) & 0\\ \frac{C_{AO,g}\left(x\right)}{\left(P_{1}-P_{AO,g}\right)} & 0 & -\frac{1}{C_{AO,g}\left(x\right)}\left(P_{AO,g}-P_{\text{out }}\right) & 0\\ 0 & \frac{1}{C_{\text{LA},g}\left(x\right)}\left(P_{1}-P_{\text{LA},g}\right) & 0 & -\frac{1}{C_{\text{LA},g}\left(x\right)}\left(P_{\text{LA},g}-P_{\text{out }}\right)\\ 0 & \frac{1}{C_{\text{LA},g}\left(x\right)}\left(P_{1}-P_{\text{LA},g}\right) & 0 & -\frac{1}{C_{\text{LA},g}\left(x\right)}\left(P_{\text{LA},g}-P_{\text{out }}\right) \end{array}\right] \end{array}, \end{array}$$

where *g* is the conductance of the gas valve, *A*(*x*, *t*) and *B*(*x*) are state and input matrices.

### 2.8. A New Volume–Elastance Equation

In hMCL applications, achieving LVV that matches both LVP and AOP characteristics presents a challenge due to the limited physiological capacitance range provided by hMCL designs. The mechanical tank used in the hMCL device does not adequately capture the low-capacitance values observed in the human body. Consequently, the volume change of the Frank-Starling Mechanism (FSM) fails to track the reference LVP and AOP signals [28, 31]. To address this limitation, a new volume–elastance equation is proposed that incorporates both the LVV and the capacitance value of the LV gas chamber. In physiological terms, LV contractility represents the instantaneous change in elastance associated with its capacitance. Therefore, the hMCL LVchamber is modeled as a gas capacitance to accurately mimic the physiological behavior of the LV muscle during contraction and relaxation. The coupling between volume and elastance is derived based on this capacitance, faithfully replicating the physiological nature of the FSM. By adopting this approach, our proposed hMCL setup can modulate the LVP in response to the LV volume stored in the LV chamber. This novel volume–elastance equation allows the proposed hMCL to not only track the targeted LVV in line with the FSM but also dynamically adjust the LVP according to the LVV.

To ensure the physiological behavior of the CVS, the system is reduced to three dynamic equations while leaving other components free in the simulation process. In physiology, most elements of the CVS are passive, except for the heart muscle, which acts as a primary actuator generating sufficient pressure and flow rate. Thus, it is crucial in this context to ensure that only the LVV is controlled to achieve FSM-driven adjustments of the proper LV contractility rate, aligning with the physiological functioning of the CVS.

In order to obtain total capacitance equation, first, left ventricular gas chamber mass can be stated as follows:

(47) $$\begin{array}{c} m_{\text{LV}},g=\rho_{\text{LV},g}V_{\text{LV},g}, \end{array}$$

where *ρ*_LV,*g*_ and *V*_LV,*g*_ are the density and volume of LV chamber. Then, derivative of Equation (47) gives us

(48) $$\begin{array}{c} {\dot{m}}_{\text{LV},g}={\dot{\rho }}_{\text{LV},g}V_{\text{LV},g}+{\dot{V}}_{\text{LV},g}\rho_{\text{LV},g}. \end{array}$$

Using Equation (5), the chain rule is applied on Equation (48) to derive the collective capacitance parameter like

(49) $$\begin{array}{c} {\dot{m}}_{\text{LV},g}={\dot{P}}_{\text{LV},g}C_{g}\left(t\right), \end{array}$$

or

(50) $$\begin{array}{c} {\dot{m}}_{\text{LV},g}={\dot{P}}_{\text{LV},g}\left(C_{\text{LV},g}+\frac{dC_{\text{LV},g}}{dP_{\text{LV},g}}P_{\text{LV}},g\right), \end{array}$$

where *C*_*g*_(*t*) is collective parameter indicating (*C*_*LV*,*g*_+*dC*_LV,*g*_/*dP*_LV,*g*_*P*_LV_, *g*) not only compliance valueof gas but it incorporates the dynamics of the liquid and gas. Now, splitting the ${\dot{V}}_{\text{LV,g}}$into differentiable parts as follows:

(51) $$\begin{array}{c} {\dot{V}}_{\text{LV},g}=\frac{dV_{\text{LV},g}}{dP_{\text{LV},g}}\frac{dP_{\text{LV},g}}{dt}. \end{array}$$

Using Equation (5) to turn the capacitance formula into the volume one, the last form equals to

(52) $$\begin{array}{c} {\dot{m}}_{\text{LV},g}={\dot{P}}_{\text{LV},g}\left(\frac{V_{\text{LV},g}}{RT}+\frac{dV_{\text{LV},g}}{dP_{\text{LV},g}}\frac{P_{\text{LV},g}}{RT}\right). \end{array}$$

Now, *C*_*g*_(*t*) can be obtained using Equation (20) like

(53) $$\begin{array}{c} C_{g}\left(t\right)=\frac{1}{RT}\left[V_{T}+\frac{2AP_{\text{LV},g}}{\rho_{l}G}-\frac{P_{\text{LV}}A}{\rho_{l}G}\right]. \end{array}$$

This calculation plays a vital role in controlling the proposed system as it enables the specific control of the LV gas section, analogous to controlling the heart muscle in physiological terms. Additionally, it is necessary to determine the dynamics of the LVV to establish the structure of the volume controller within the hMCL.

In the proposed hMCL, the numerical model is obtained using the state–space representation given in Equation (46). The equations of motion of the CVS is obtained using physiological signals *x*(*t*) at the same equation. However, the CVS order must be reduced to three equations in order to implement the new volume–elastance formula given in Equation (53). The reduced order CVS modelis now rewritten as follows:

(54) $$\begin{array}{c} \begin{array}{c} {\dot{P}}_{1}=g_{1}\left(P_{c}-P_{1}\right)\alpha_{1}-g_{2}\left(P_{1}-P_{\text{LV},g}\right)\alpha_{1}\left.\;\right]\\ {\dot{P}}_{\text{LV},g}=g_{2}\left(P_{1}-P_{\text{LV},g}\right)\alpha_{g}-g_{3}\left(P_{\text{LV},g}-P_{\text{out }}\right)\alpha_{g}\\ {\dot{V}}_{\text{LV},l}=-\frac{A}{\rho_{l}G}\left[g_{2}\left(P_{1}-P_{\text{LV},g}\right)\alpha_{g}-g_{3}\left(P_{g}-P_{\text{out }}\right)\alpha_{g}\right], \end{array} \end{array}$$

where *αg* is collective volume–elastance formula. This last form only includes heart muscle analogy of the present hMCL. It is important to note that the proposed hMCL should only be controlled using the reference LVV signal in order to satisfy the coupling both the FSM and LV contractility rate in a physiological manner. Therefore, it is sufficient to design discrete feedback linearization control guaranteeing stability for simulating the physiological CVS. In order to drive the proposed hMCL, there are two reference signals needs to be defined as LVV (*V*_ref_) and balance tank pressure one (*P*_1,ref_). The *V*_ref_ is established using the maximum and minimum values of LVV required to provide the FSM. On the other hand, *P*_1,ref_ is selected as unit step signal which can easily be adjusted using a coefficient that shows the targeted pressure value in the balance tank.

### 2.9. Stability Analysis

The continuous model, Equation (54), is discretized using Euler forward difference equation. In this study, discrete feedback linearization controller design is performed in order to track the reference LVV given within the range of physiological conditions using Lyapunov stability theorem similar by İşcan et al. [37].

### 2.10. Error System Dynamics

The main objective of the hMCL device is to track a reference LV volume signal, *V*_ref_ (*k*) defining physiological CVS. Thus, we define LVV error

(55) $$\begin{array}{c} e_{\text{LV}}\left[k\right]=V_{\text{ref}}\left[k\right]-V_{\text{LV},l}\left[k\right], \end{array}$$

and the pressure

(56) $$\begin{array}{c} e_{p1}\left[k\right]=P_{1},\text{ref}\left[k\right]-P_{1}\left[k\right], \end{array}$$

with *V*_*ref*_[*k*] and *P*_*ref*_[*k*] the reference LVV and the pressure signals satisfying human CVS hemodynamics. Discretizing LVV from Equation (54) yields

(57) $$\begin{array}{c} V_{\text{LV},l}\left[k+1\right]=V_{\text{LV},l}\left[k\right]+\Delta t\left(f_{\text{LV}}g_{2}\left[k\right]V_{11}\left[k\right]-g_{3}\left[k\right]V_{12}\left[k\right]\right), \end{array}$$

where

(58) $$\begin{array}{c} \begin{array}{c} f_{\text{LV}}=-\frac{A}{\rho_{l}G}dt\\ V_{11}\left[k\right]=\left(P_{1}\left[k\right]-P_{\text{LV},g}\left[k\right]\right)\alpha_{g}\left[k\right]\\ V_{12}\left[k\right]=\left(P_{\text{LV},g}\left[k\right]-P_{\text{out }}\left[k\right]\right)\alpha_{g}\left[k\right]. \end{array} \end{array}$$

Substituting Equation (57) into the LVV error equation yields the reference tracking error dynamics as follows:

(59) $$\begin{array}{c} e_{\text{LV}}\left[k+1\right]=V_{\text{ref}}\left[k+1\right]-V_{\text{LV},l}\left[k\right]-\Delta t\left(f_{\text{LV}}g_{2}\left[k\right]V_{11}\left[k\right]-g_{3}\left[k\right]V_{12}\left[k\right]\right). \end{array}$$

Similarly, steps are applied to the discrete pressure difference equation from Equation (54) as follows:

(60) $$\begin{array}{c} P_{1}\left[k+1\right]=P_{1}\left[k\right]+f_{p}\left(g_{1}\left[k\right]V_{21}\left[k\right]-g_{2}\left[k\right]V_{22}\left[k\right]\right), \end{array}$$

where

(61) $$\begin{array}{c} \begin{array}{c} f_{p}=\Delta t\\ V_{21}\left[k\right]=\left(P_{c}\left[k\right]-P_{1}\left[k\right]\right)\alpha_{1}\left[k\right]\\ V_{22}\left[k\right]=\left(P_{1}\left[k\right]-P_{\text{LV},g}\left[k\right]\right)\alpha_{1}\left[k\right]. \end{array} \end{array}$$

Substituting Equation (60), now, into the pressure error, the tracking error dynamics becomes

(62) $$\begin{array}{c} e_{p1}\left[k+1\right]=P_{1,\text{ref}}\left[k+1\right]-P_{1}\left[k\right]-f_{p}\left(g_{1}\left[k\right]V_{21}\left[k\right]-g_{2}V_{22}\left[k\right]\right), \end{array}$$

We are now, ready to state the controller to achieve the reference tracking of LV signals.

**Theorem 1 .**

The pneumatic valve control signals

(63) $$\begin{array}{c} g_{1}\left[k\right]=\frac{A_{e,P1}e_{p_{1}}\left[k\right]-P_{1,\text{ref}}\left[k+1\right]+P_{1}\left[k\right]-f_{p}V_{22}g_{2}\left[k\right]}{-f_{p}V_{21}\left[k\right]}, \end{array}$$

(64) $$\begin{array}{c} g_{2}\left[k\right]=\frac{C_{1}}{C_{g}}, \end{array}$$

(65) $$\begin{array}{c} g_{3}\left[k\right]=\frac{A_{e,\text{LV}}e_{\text{LV}}\left[k\right]-V_{\text{ref}}\left[k+1\right]+V_{\text{LV},l}\left[k\right]-f_{\text{LV}}V_{11}\left[k\right]g_{2}\left[k\right]}{-f_{\text{LV}}V_{12}\left[k\right]}, \end{array}$$

with |*A*_*e*,*P*_1__ < 1| and |*A*_*e*,*LV*_ < 1| gains, applied to the hMCL system ensures the stability of the closed-loop system.

**Proof**

The quadratic tracking error is selected as the Lyapunov function candidate both for the pressure and LVV

(66) $$\begin{array}{c} L\left[k\right]=L_{P}\left[k\right]+L_{\text{LV}}\left[k\right]=e_{P_{1}}{\left[k\right]}^{2}+e_{\text{LV}}{\left[k\right]}^{2}, \end{array}$$

with *L*_*P*_[*k*]=*e*_*P*_1__[*k*]^2^ and *L*_*LV*_[*k*]=*e*_*LV*_[*k*]^2^. The difference equation for *L*_*i*_[*k*] for *i*={*P*_1_, *LV*} is

(67) $$\begin{array}{c} L_{i}\left[k+1\right]-L_{i}\left[k\right]=e_{i}{\left[k+1\right]}^{2}-e_{i}{\left[k\right]}^{2}. \end{array}$$

By substituting the control signals Equations (63–65) into the pressure and the LVV error dynamics given in Equations (62) and (59), it can be shown by direct substitution that

(68) $$\begin{array}{c} e_{i}\left[k+1\right]=A_{e,i}e_{i}\left[k\right]. \end{array}$$

Thus,

(69) $$\begin{array}{c} L_{i}\left[k+1\right]-L_{i}\left[k\right]=\left(A_{e,i}^{2}-1\right)e_{i}{\left[k\right]}^{2}\leq 0, \end{array}$$

since |*A*_*e*,*i*_ < 1|.

Therefore, the nonincreasing Lyapunov functions guarantee the boundedness of *e*_*i*_[*k*] reference tracking signals and observing the relationship between the gas pressure and liquid volume of ${\dot{P}}_{LV,g}=-\rho_{l}G{\dot{V}}_{LV,l}/A$ one can deduce the boundedness of *P*_*LV*,*g*_. This concludes the stability of the overall system.

**Remark 1 .**

We have used mainly the LVV reference signal to generate left ventricular pressure and mass flow rate signals from the hMCL device, satisfying the FSM.

To match physiological response of CVS with the proposed hMCL, the capacitance values of aorta and LA must be satisfied. In order to control the capacitance of the aortic and left atrium tank, the same logic must be followed as given above. However, Equation (54) must be rewritten representing both the aortic and left atrium capacitance formulation which are given in both Equations (32) and (40).

## 3. The hMCL Verification

In this study, all experiments were conducted using both the Matlab 2022a Simulink Platform and the proposed the hMCL. A comparison and validation of the results were then carried out to ensure their consistency with the physiological response of the CVS.

First, the numerical and hMCL experiments were conducted to demonstrate the results, including healthy cases, in terms of pressure, volume, and both active and passive pressure of the LV. The physiological limits were assessed to determine the similarity between our model and the physiological model described in the literature. Second, the proposed hMCL was tested by varying the resistance values to simulate aortic stenosis and systemic abnormalities, achieved by adjusting the orifice area of the pipelines. This allowed for the evaluation of time response. Third, the different heart rate references were applied to assess the cardiac output, in comparison to physiological studies documented in the literature.

**Figure 3:**
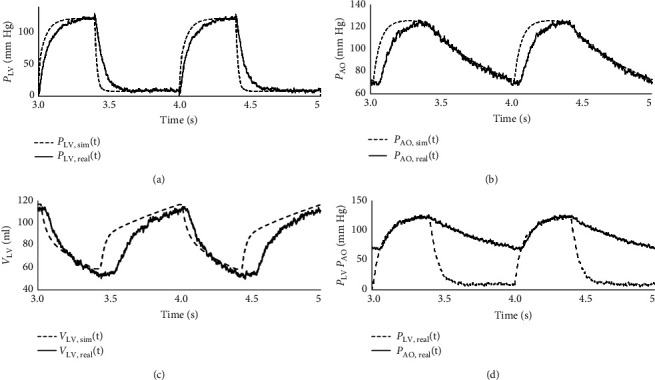
Results of the numerical and hMCL: (a) LVP, (b) AOP, (c) LVV, and (d) only hMCL.

Figure 3 demonstrated a comparison between the proposed hMCL and numerical experiment results, focusing on the LVV, as well as the AOP and LVP.

In Figure 3, Figure 3(a) and (Figure 3(b)) represented the hMCL measurement of LVP and AOP, respectively. It is noteworthy that the numerical CVS responses closely matched the hMCL responses. However, there were some signal distortions due to sensor and electronic component limitations. The overall median error between the hMCL and numerical simulation was calculated as 3.01 ± 11.23 mm Hg. In Figure 3(c), the LVV responses were presented to compare the hMCL and numerical data. Achieving control over the volume changes in the left ventricle chamber led to more accurate results, with a standard deviation of 1.43 ± 5.24 mm Hg, demonstrating a close resemblance between the numerical and hMCL data.

**Figure 4:**
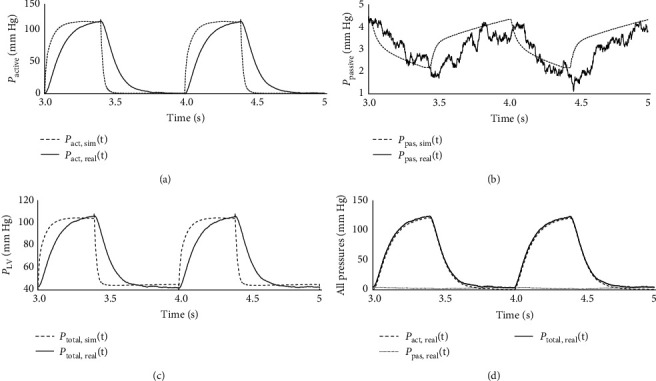
Results of active–passive pressures. (a) active LVP, (b) passive LVP, (c) total LVP, and (d) hMCL measurements.

The active and passive pressures of LV were demonstrated in Figure 4.

Figure 4 illustrated the numerical and hMCL outcomes of the LVP. The passive pressure was determined by considering the volume changes of the chamber in the actual experiment, leading to more precise results. On the other hand, the active pressure was derived using the proposed volume–elastance equation. The findings unmistakably demonstrate that the overall pressure in the left ventricle can be regulated by controlling either the pressure in the gas chamber or its active component, aligning with the physiological perspective. The error between both numerical and hMCL experiment were performed at the rate of 0.34 ± 4.36 and 8.18 ± 7.15 mm Hg for passive and active pressures.

**Figure 5:**
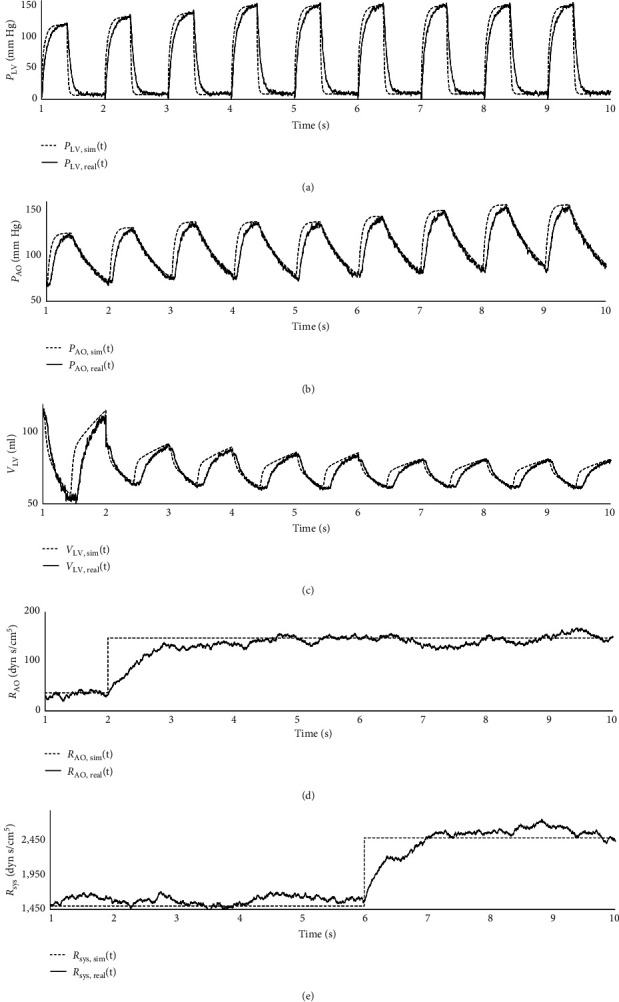
The results of aortic stenosis and SVR response: (a) LVP, (b) AOP, (c) LVV, (d) aortic resistance, and (e) SVR.

After these tests accomplished, cardiovascular dysfunctions were tried to observe on the numerical and hMCL experiments.  Figure 5 demonstrated these results by changing resistance values corresponding with the aortic stenosis and systemic vascular resistance (SVR).

In Figure 5, the pressure, resistance, and volume values were measured from the numerical and hMCL.  Figure 5(a) and Figure 5(b) represented the pressure response under the aortic stenosis condition which means that aortic resistance increased while constant heart rate and reference volume in left ventricle chamber. After passing 6 s, the SVR was increased in order to obtain the time response of the proposed hMCL. The pressure value increased when flow rate of left ventricle decreased. At the hMCL experiment, it gave the correlation at the rate of 14.15 ± 22.12 mm Hg, 4.29 ± 13.22 mm, and 1.04 ± 1.43 mm Hg s/ml. The same test with systemic performance also caused differences at the rate of 12.41 ± 18.03 mm Hg, 4.91 ± 7.54 mm/ml, and 0.11 ± 0.23 mm Hg s/ml.

**Figure 6:**
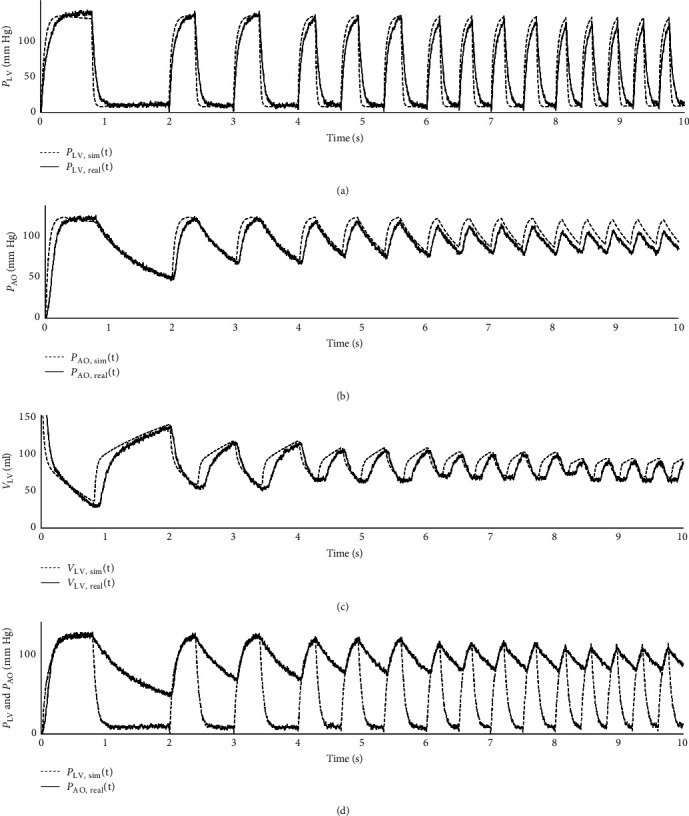
The results of different heart rate: (a) LVP, (b) AOP, (c) LVV, and (d) the hMCL LVP and AOP.

The last test to prove the physiological imitation with hMCL was performed at the different heart rate conditions in the range of 30–120 bpm. The results were illustrated in Figure 6.

In Figure 6. the numerical and hMCL results were given containing the different heart rates and their corresponding volume change in left ventricular chamber. It was noteworthy that the numerical results were consistent at every heart rate for both volume and pressure values of left ventricle. However, hMCL experiments clearly showed that the volume change of left ventricle decreased when heart rate increased. At the same time, the pressure values were not significantly affected by the heart rate deviation in real-time experiment.

## 4. Discussion

This paper presents a novel design and control approach for hMCL, incorporating a new volume/elastance equation to simulate the FSM in the CDS. The methodology is subjected to both mathematical and physiological considerations.

From a mathematical standpoint, we proposed a discrete nonlinear system dynamic model along with its corresponding controller structure. To the best of our knowledge, there is currently no established controller rule that guarantees stability for gas valve dynamics in the discrete time domain. In contrast to the previous studies, our discretized feedback linearization controller design focuses exclusively on the gas section of the left ventricle model. The proposed LVV–E plays a crucial role in providing physiological relevance to the hMCL, as they enable control over only the LV gas chamber, thereby activating the left ventricular muscle in physiological manner. The remaining state variables of the hMCL are left unconstrained, aligning with physiological conditions.

Numerical and hMCL simulations are performed to assess the reliability between the FSM and LVC.  Table 2 presents the performance comparison between the numerical and hMCL results, offering insights into the effectiveness of our approach.

Table 2 demonstrates a close correspondence between the numerical and hMCL results. Various factors contribute to the observed discrepancies, including the discretization process applied to the mathematical model, parameters of the Kalman filter, sensor noise, noise introduced by the microcontroller's ADC module, and limitations in the mechanical structure. The discretization is achieved using Euler forward approximation which means that the representation of the nonlinear dynamics of CVS is degraded. However, this approximation paves the way for implementation of controller structure on STM32 microcontroller which has higher sampling period. The Kalman filter parameters (*Q*, *R*) possess the direct effect on suppressing the noises on both process and measurements. These values are kept to be constants in real-time application causing some differences between numerical and hMCL experiments. Especially for ADC-induced noise, it limits the resolution of the measured pressures from the hMCL. If the sampling period of the ADC module increases, the resolution of the pressure value decreases. This obligates to find the optimized values on the selection of the sampling period of the ADC which reduces the measured pressure quality. It is particularly important to assess the performance of the hMCL in light of these mechanical limitations, as the resistance and capacitance values may deviate from their real counterparts due to the three-dimensional shape of the device. Although the Kalman filter helps mitigate these issues within the controller algorithm and nonlinear model, achieving a one-to-one match in such systems is not always feasible.

In Table 3, the proposed hMCL results are illustrated under three cases: physiological left ventricle, aortic stenosis, and systemic vascular change compared to the other studies in the literature.

In Table 3, the proposed hMCL results are compared to other studies. Especially in the evaluation of CO, our results have correlation with 4.86 ± 1.04 l/min except Jansen-Park et al. [25] study due to utilization of LVAD. LVAD mainly causes variations in LVQ supplied by hMCL device. Mean LVP happens 60.28 ± 6.38 mm Hg since Packy et al. [32] set heart rate as 75 bpm unlike we have 60 bpm test condition. Mean AOP has the standard deviation of 83.13 ± 12.38 mm Hg. In Gregory et al. [24] study, AOP is higher than the proposed one because the pneumatic actuator of their study is directly taken pressure reference control, unlike our study utilizes only LVV reference. In physiology, the CVS cannot regulate the LVV with respect to the LVP since the FSM states that only preload can change the CO which means that the regulation is initiated from LVV requirement of the body. SVR case is achieved in close to the other studies with the standard deviation of 1703.3 ± 370.18 dyn s/cm^5^. For the mean aortic resistance, only Salesch et al. [28] reported scalar value with the difference of 10.18 dyn s/cm^5^. Systemic and aortic resistances depend on the mechanical component and manual valve which are different from the proposed hMCL. However, they are acceptable to meet the criteria of the proposed hMCL since the standard deviation and mean error happen at the rate of <10.

For the AS case, only Rapp et al. [31] gave the some graphical results. However, their device structure is different from our one, so, resistance; LVP and AOP are changed and compared to our study. Their setup consists of two sections including aortic and LV sections. On the other hand, both sections are controlled tracking reference AOP and LVP while the proposed hMCL ones are set to be free. Besides, the time response of aortic stenosis in their study corresponds to our results especially in terms of AOP and LVP. On the other hand, our previous work has strong correlation with the present study due to the utilization of the same aortic valve model [36].

In the case of increasing SVR, mean SVR value is matched with Colacino et al.'s [18] result.

The mean error rate is equal to 145.66 dyn s/cm^5^ and is caused by different pipeline resistance and squeeze points. Their pipeline has smaller diameter leading to smaller resistance value. The other parameters are higher than our ones that we have obtained. Several reasons may cause these differences. Colacino et al. [18] study utilize LVV reference signal generated by pressure reference of LV. Also, mechanical pump generates the volumetric flow rate to supply the system in their study. Capacitance values of the tanks are different which means that they have different pressurizing range in their setup. Besides, aortic stenosis is present with systemic vascular increment in our study; therefore, we may have different results compared to the other studies.

Finally, heart rate change is important matter to discuss about the imitation performance of the proposed hMCL with its discrete nonlinear system dynamic modeling. LVV is decreased when the heart rate increase since the FSM dictates the transfer rate depending on preload values of the heart [38]. In physiology, increasing heart rate yields increasing CO. However, at the high-heart rates like >150 bpm, the stroke volume approaches the steady-state value on the CVS. It is important to satisfy this property since the CV physiology behaves different responses as well as the FSM. In hMCL results, although this constraint is not broken when increasing heart rate, real-time experiments showed that LVV is decreased with increasing heart rate. Stroke volume error value happens with the rate of 9.25 ml at the heart rate between 60 and 120 bpm with respect to Guyton and Hall's [38] data. On the other hand, the proposed hMCL can replicate the FSM even in the high-heart rate which is more consistent with the CV physiology.

The validity and innovations of the presented study also need to be discussed. Particularly, in clinical applications, the complex and invasive design of the presented hMCL is expected to provide a significant advantage in abnormal conditions. Clinical validation tests, especially testing with AOP and heart rate periods, should be conducted, and if measurements of maximum elastance values are possible, tests should be performed for validation. Due to the impossibility of direct left ventricular pressure and volume measurements, the performance of the presented hMCL can be observed by looking at AOP and ECG values. On the other hand, for future studies, obtaining and validating the prediction results of the nonlinear cardiovascular model constructed using AOP and ECG signals is also important.

## 5. Conclusions

In this study, we have introduced a novel design for an hMCL that incorporates a nonlinear discrete equation of motion and discrete feedback linearization control. The proposed study makes several significant contributions to the field. First, it successfully replicates the FSM that adheres to LVC criteria. Second, it formulates the nonlinear dynamics of the CVS discretely, aligning it with a proposed in vitro setup. It also ensures the stability of the closed-loop hMCL in the discrete domain, facilitating real-time applications. Besides, it eliminates the need for predefined functions or physiological data to emulate CVS. Last, it introduces a controlled parameter while leaving others free, enhancing the realism of CVS simulations. These contributions collectively advance our understanding and capabilities in CV modeling and control. A key contribution of this research is the proposal of a new capacitance formula, which allows for a more realistic representation of the electrical excitation of the left ventricle compared to conventional methods. Especially for the LVV–E, the proposed capacitance equation enables us to create direct relation between LVV and LVC resulting in the replication of the FSM. This gives us an opportunity to control and model the CVS dynamics satisfying the FSM with respect to the physical in vitro setup. As a result, the hMCL is capable of accurately emulating the FSM by controlling the volumetric rate of the left ventricle across a range of heart rates (30 to 120 bpm), volumetric flow rates (0–5l /min),and pressures (70–150 mm Hg). Physiologically, the values provided above allow testing of the CVS in both normal and abnormal patients. The ability to adjust heart rate along with pressure and flow cycles enables the modeling of various abnormal conditions. Furthermore, because the FSM can generate physiological responses under these conditions, the presented hMCL has been observed to produce meaningful results in the clinical tests. Conclusions section should clearly explain the main findings and implications of the work, highlighting its importance and relevance.

The conducted experiments have provided validation of the hMCL's capability to simulate critical cardiovascular conditions, including aortic stenosis, changes in systemic resistance, and variations in heart rate. Remarkably, the hMCL achieves these simulations solely based on the left ventricular volume reference, demonstrating its effectiveness in replicating both normal and abnormal cardiovascular conditions. Providing FSM under diseased conditions is crucial for cardiovascular studies. Along with ensuring FSM, preliminary diagnosis of various diseases and the observation of possible pressure and flow changes become particularly important for medical education and prediction. Verification of critical cardiovascular conditions has been performed in a repeatable manner by comparing them with the other studies, without disrupting FSM.

Moving forward, our future studies will focus on addressing the mechanical limitations associated with the hMCL, particularly the dynamics of opening and closing valves (aortic and mitral) that impact resistance. Particularly, the mechanical structure is the most crucial factor affecting performance in the in vitro setup. In the physiological CVS, the opening and closing of valves cause instant changes in resistance, and the inability of the proposed method to capture this change leads to a decrease in performance. On the other hand, for FSM to be implemented more accurately physiologically and clinically, the measurement of ECG and the presence of heart rate, contraction, and relaxation times are aimed, and hMCL is driven in this way. This will enhance physiological accuracy, facilitating the identification of unmeasurable physiological parameters for the clinical studies. To overcome this challenge, we will develop an estimation algorithm that employs a neural network controller structure to predict real-time resistance values in the mock system. Furthermore, we aim to enhance the ECG-driven volume reference trajectory by incorporating additional parameters such as heart rate, contraction/relaxation periods, and the strength of contraction and relaxation, leveraging the interpretation of ECG signals.

In conclusion, the proposed hMCL system has demonstrated high performance in accurately simulating the physiological properties of the cardiovascular system, with a particular focus on the FSM, under both normal and abnormal conditions. The use of the presented hMCL can provide an insight into the estimated values of CVS parameters that cannot be measured noninvasively. Furthermore, in medical education, modeling CVS noninvasively and simulating various disease conditions will create a clinical application area that doctors/experts can easily use in decision-making and diagnosis processes. Its ability to replicate a wide range of cardiovascular scenarios makes it a valuable tool for further understanding and investigating the cardiovascular dynamics.

## Figures and Tables

**Table 1:** Symbols table.

Parameter	Name	Value	Unit
*P*_*i*_	Total pressure for “*i*” tank		mm Hg
*P*_*i*,*g*_	Gas pressure for “*i*” tank		mm Hg
Δ*P*_*i*_in__	Inlet pressure difference		mm Hg
Δ*P*_*i*_out__	Outlet pressure difference		mm Hg
*P*_*c*_	Compressor pressure		mm Hg
*P*_*atm*_	Atmospheric pressure		mm Hg
*V*_*i*,*g*_	Gas volume for “*i*” tank		ml
*V*_*i*,*l*_	Liquid volume for “*i*” tank		ml
*V*_*t*_	Total tank volume		ml
*m*_*st*,*i*_	Tank mass storage for “*i*” index		kg
*m*_*i*_	Transferred mass between “*i*”and “*i* + 1”tank		kg
*m*_*i*,*g*_	Gas mass for “*i*”tank		kg
*ρ*_*i*_	Gas density for “*i*”tank		kg/ml
*ρ*_*l*_	Liquid density for blood		kg/ml
*C*_*i*,*g*_(*t*)	Gas compliance parameter for “*i*”tank		kg/mm Hg
*C*_*g*_(*t*)	Control collective compliance parameter		kg/mm Hg
*α*=1/*C*_*i*_	Elastance for “*i*”tank		kg/mm Hg
*g*_*i*_	Valve conductance between “*i*” and “*i* + 1” tank		kg/mm Hg s
*A*_*e*,LV_	Control coefficient for left ventricular volume		–
*A*_*e*,LV_	Control coefficient for Tank 1 pressure		–
*R*	Ideal gas constant		mm Hg ml/kg K
*T*	Temperature	273 + 23	K
*D*	Pipeline diameter	0.014	m
*L*	Pipeline length	0.35	m
*μ*	Liquid viscosity	1.0016 · 10^−3^	–
*A*	Tank area	0.002	m^2^
*G*	Gravity	9.81	m/s^2^
*g*_*P*_	Pipeline conductance rate	0.00027	ms
*g*_*M*_	Physical mitral conductance rate	0.00027	ms
*g*_*AO*_	Physical aortic conductance rate	0.00027	ms
*g*_*SYS*_	Physical systemic conductance rate	0.00027	ms
*g*_*ao*_	Physical aortic valve conductance rate	0.1504 · 10^−4^	ms
*g*_*m*_	Physical aortic valve conductance rate	0.2506 · 10^−4^	ms
*θ*_*m*_(*t*)	Time-varying mitral opening rate		
*θ*_*AO*_(*t*)	Time-varying aortic opening rate		
*C*_*i*_(0)=*A*/*g*	Initial physical tank compliance	2.0387 · 10^−3^	ms

**Table 2:** The numerical and hMCL results.

@60 bpm	PV		AS		SVR	
The proposed	Numerical	hMCL	Numerical	hMCL	Numerical	hMCL
*P*_LV,max_ (mm Hg)	131.22	133.78	153.52	156.08	156.14	157.13
*P*_LV,min_ (mm Hg)	10.23	7.61	13.87	10.42	12.58	14.27
*P*_AO,max_ (mm Hg)	129.11	130.27	141.19	139.37	153.18	151.35
*P*_AO,min_ (mm Hg)	74.64	72.48	78.33	75.17	89.77	87.41
*V*_LV,max_ (ml)	118.56	116.23	97.44	99.76	91.06	92.32
*V*_LV,min_ (ml)	50.04	46.41	54.24	51.14	56.61	56.13
*SV*_mean_ (ml)	64.52	67.82	38.19	41.66	31.39	34.65
*CO*_mean_ (ml)	4.64	4.86	2.74	2.99	2.29	2.49
*R*_AO,mean_ (dyn s/cm^5^)	34	30.14	144	140.53	144	140.53
*R*_sys,mean_ (dyn s/cm^5^)	1500	1703.38	1500	1703.38	2,500	2919.1

**Table 3:** Comparison table under physiological ventricle (PV), aortic stenosis (AS), and systemic vascular resistance (SVR) change.

		*P*_LV,mean_	**P**_AO,mean_	**V**_LV,max_	**V**_LV,min_	SVmean	**C** **O**_mean_	**R**_AO,mean_	**R**_sys,mean_
PV	The proposed hMCL	60.28	83.13	116.23	46.41	67.82	4.86	30.14	1703.38
	Packy et al. [32]	53.9	73.4	–	–	67.00	5.03	–	1346.2
	Bardi et al. [33]	–	94.13	–	–	–	4.47	–	–
	Vignali et al. [29]	–	–	–	–	–	4.71	–	–
	Salesch et al. [28]	–	–	–	–	–	–	40.32	1333.2
	Gregory et al. [27]	62.5	96	135	52	83	4.98	–	1,400
	Jansen-Park et al. [25]	–	91	–	–	50	3.816	–	1728
	Gregory et al. [24]	–	110	–	–	72.2	5.2	–	–

AS	The proposed hMCL	74.87	118.13	99.76	51.14	41.66	2.99	140.53	1703.38
	Rapp et al. [31]	54.13	61.65	–	–	–	–	28.8	1548
	Iscan and Yesildirek [36]	78.44	112.47	92.18	47.13	34.11	2.45	115.2	1598.73

SVR	The proposed hMCL	80.45	125.48	92.32	56.13	34.65	2.49	140.53	2919.1
	Colacino et al. [18]	–	160	150	70	80	4.8	–	2773.44

## Data Availability

The data sets used and/or analyzed during the current study are available from the corresponding author on reasonable request.

## Supplementary Materials

Supplementary Materials The video representation of the proposed hMCL device is prepared and the following link can be used to watch the whole procedure related to how we design and implement our device (Video link: https://youtu.be/ngUQdHZ1iaQ).
