# Supplementary material for: A New Cardiovascular Mock Loop Driven by Novel Active Capacitance in Normal and Abnormal Conditions
Source: Appl Bionics Biomech. 2023 Oct 27;2023:2866637. doi: 10.1155/2023/2866637 (PMC10624551; doi:10.1155/2023/2866637)
Supplement: Supplementary Materials — The video representation of the proposed hMCL device is prepared and the following link can be used to watch the whole procedure related to how we design and implement our device (Video link: https://youtu.be/ngUQdHZ1iaQ). [file 2866637.f1.docx]

Our device can be seen in the following link:

https://youtu.be/ngUQdHZ1iaQ
